# Artificial Intelligence Application in a Case of Mandibular Third Molar Impaction: A Systematic Review of the Literature

**DOI:** 10.3390/jcm13154431

**Published:** 2024-07-29

**Authors:** Hassan Ahmed Assiri, Mohammad Shahul Hameed, Abdullah Alqarni, Ali Azhar Dawasaz, Saeed Abdullah Arem, Khalil Ibrahim Assiri

**Affiliations:** Department of Diagnostic Science and Oral Biology, College of Dentistry, King Khalid University, P.O. Box 960, Abha City 61421, Saudi Arabia; mhmead@kku.edu.sa (M.S.H.); aawan@kku.edu.sa (A.A.); adwasaz@kku.edu.sa (A.A.D.); sareem@kku.edu.sa (S.A.A.); alasery@kku.edu.sa (K.I.A.)

**Keywords:** impacted tooth, mandibular third molar, artificial intelligence, panoramic radiography, cone beam computed tomography

## Abstract

**Objective:** This systematic review aims to summarize the evidence on the use and applicability of AI in impacted mandibular third molars. **Methods:** Searches were performed in the following databases: PubMed, Scopus, and Google Scholar. The study protocol is registered at the International Platform of Registered Systematic Review and Meta-analysis Protocols (INPLASY202460081). The retrieved articles were subjected to an exhaustive review based on the inclusion and exclusion criteria for the study. Articles on the use of AI for diagnosis, treatment, and treatment planning in patients with impacted mandibular third molars were included. **Results:** Twenty-one articles were selected and evaluated using the Scottish Intercollegiate Guidelines Network (SIGN) evidence quality scale. Most of the analyzed studies dealt with using AI to determine the relationship between the mandibular canal and the impacted mandibular third molar. The average quality of the articles included in this review was 2+, which indicated that the level of evidence, according to the SIGN protocol, was B. **Conclusions:** Compared to human observers, AI models have demonstrated decent performance in determining the morphology, anatomy, and relationship of the impaction with the inferior alveolar nerve canal. However, the prediction of eruptions and future horizons of AI models are still in the early developmental stages. Additional studies estimating the eruption in mixed and permanent dentition are warranted to establish a comprehensive model for identifying, diagnosing, and predicting third molar eruptions and determining the treatment outcomes in the case of impacted teeth. This will help clinicians make better decisions and achieve better treatment outcomes.

## 1. Introduction

Artificial intelligence (AI) refers to the application of machines and technology to perform human tasks. This technology can learn and apply knowledge to perform specific tasks in different fields [1]. Barr and Feigenbaum described AI as the branch of computer science that focuses on creating a computer system that demonstrates traits associated with intelligence, such as language comprehension, learning, reasoning, and problem-solving [2]. AI techniques are being applied in medicine for the diagnosis, prognostic assessment, and predictability of diseases. According to a recent systematic review, AI has demonstrated the ability to automatically detect coronary artery calcification, cerebral microhemorrhages, diabetic retinopathy, and breast or skin cancer [3]. Several terms related to AI, such as machine learning, neural networks, and deep learning, have been used in the literature [4]. Machine learning is a subset of AI that uses algorithms to predict outcomes from data sets. Neural networks are a part of AI that use algorithms to predict outcomes based on data. The goal of machine learning is to make it easier for machines to learn from data so that they can solve problems without human intervention. Deep learning is primarily a component of machine learning, wherein the input data are analyzed using a deep neural network of many computational layers. Building a neural network with automatic pattern recognition to improve feature identification is the goal of deep learning [5].

AI has been integrated into various aspects of dentistry, including the diagnosis and treatment of impacted mandibular third molars. A study by Pauwels et al. [6] compared the diagnostic performance of convolutional neural networks (CNNs) with the performance of human observers for the detection of simulated periapical lesions on a periapical radiograph. In addition, Yang et al. [7] used deep learning for the automated detection of jaw cysts and tumors in panoramic radiographs. Mertens et al. [8] conducted a randomized clinical trial to investigate the impact of AI-based diagnostic support software for detecting proximal caries in bitewing radiographs. Computed tomography (CT) is the gold standard for quantifying bone density using the Hounsfield unit (HU). However, it has the disadvantage of a high radiation dose [9], resulting in the use of cone beam CT (CBCT) for bone density assessments [10]. Several studies have reported using AI for bone density assessments during preimplant placement, bone loss in periodontal diseases, and bone loss in the oral and maxillofacial regions [11,12,13]. The impacted mandibular third molar is expected to erupt between the ages of 17 and 21 years [14]. However, it is prone to impaction due to systemic or local diseases that prevent the tooth from erupting in the functioning position at the expected time and is considered the most frequently impacted tooth [15]. An inadequately detected and treated mandibular third molar impaction can present several clinical consequences [16]. AI has been applied to diagnose and evaluate a mandibular third molar impaction; AI models have been developed and applied to predict the difficulty of the extraction of impacted mandibular third molars, providing valuable information to clinicians to determine the appropriate treatment approach [17]. In addition, AI measurements of the molar angulation have been used to predict third molar eruption, aiding treatment planning and decision-making [18]. Radiographic imaging plays a fundamental role in evaluating impacted mandibular third molars. Therefore, AI algorithms have been developed to analyze panoramic radiographs and predict the eruption status and angulation of mandibular third molars [17]. Orhan et al. [19] used cone beam computed tomography (CBCT) AI to determine mandibular third molar positions.

In addition to diagnostic and treatment planning, AI has been applied in postoperative management to predict postoperative complications and consequences, such as facial swelling after the extraction of impacted mandibular third molars, thereby providing better patient management and required care [17]. However, the extent of AI application in cases of impacted third molars remains unclear. Therefore, the aim of this systematic review was to summarize the evidence on the use and applicability of AI in impacted mandibular third molars.

## 2. Methodology

This review was conducted according to the Preferred Reporting Items for Systematic Review and Meta-Analysis (PRISMA) and the Center for Reviews and Dissemination guidelines for conducting a systematic review in health care [20]. The study protocol is registered at the International Platform of Registered Systematic Review and Meta-analysis Protocols (INPLASY202460081) and is available at the discretion of the corresponding author. The review question was designed according to the PICO element (Population or Problem, Intervention or Exposure, Comparison, Outcome) for studying interventions in the health sciences.

### 2.1. Specification of the Problem

What is the application and use of AI in patients with third molar impaction?

P: patients with third molar impaction;

I: AI;

C: comparison with other tools, if applicable;

O: uses and applicability of AI.

### 2.2. Inclusion and Exclusion Criteria

The inclusion criteria were as follows: studies reported in English and Spanish; those describing AI; those conducted to investigate the mandibular third molar; and original studies, including randomized clinical trials. Reviews, studies investigating other dental aspects, technical reports, and studies reported in other languages were excluded from this review. Language restriction was carried out as, during the search, we did not find studies that met the whole inclusion and exclusion criteria in other languages.

### 2.3. Search Strategy

After applying the variables of the research question and specifying the criteria to be applied for the selection of articles in the databases, a search strategy was established based on the use of key terms, logical operators (AND, OR), and search filters for the databases PubMed, Scopus, and Google Scholar (Table 1). A series of studies was obtained, and the research criteria were applied to determine their eligibility and inclusion for analysis. The search was guided by the medical descriptors described earlier and indexed to the database of medical terms (MeSH) and natural language described by the National Institutes of Health (NIH; impacted tooth, mandibular, third molar, impaction, and artificial intelligence), which were linked by the Boolean operator AND. The filters and specific terms were combined with the topics used in each search to gain access to the largest number of articles that were relevant to the research and met the research criteria (Table 1).

### 2.4. Retrieval of Studies from the Databases

**Figure 1:**
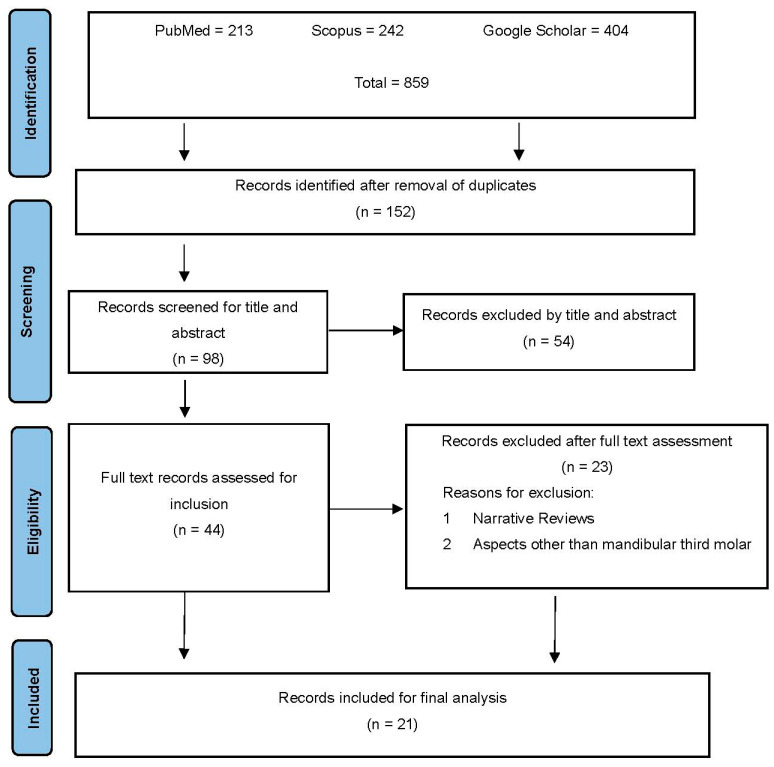
PRISMA chart illustrating the search process.

The results of the search routes and the screening of articles are represented in the flow chart (Figure 1) according to the guidelines of the PRISMA methodology [20]. Three databases (PubMed, Scopus, and Google Scholar) were searched to retrieve studies related to the objective of our research that were published from January 2019 up to January 2024. The resultant studies were subjected to duplicate checks by two authors (HA, MHS) using the RefWorks database^®^ (version 23.1). Three authors (HA, MSH, SA) screened the studies for the relevant titles and abstracts. Duplicate articles were discarded, and the titles and abstracts were examined after applying the research criteria to each article. Twenty-one articles were selected from the search results for final analysis (Figure 1).

### 2.5. Data Collection

Data from the 21 studies were extracted for further analysis based on specific criteria listed in Table 2.

### 2.6. Evaluation of the Risk of Bias

The Scottish Intercollegiate Guidelines Network (SIGN) method was used to evaluate bias in the included studies [40]. This method of evaluating the level of scientific evidence aided in simplifying the risk of bias assessment and determining the quality of the articles in the current study (Table 3). The grading system of the SIGN tool was assigned to each study where 2+ indicated a well-performed study with a low risk of bias, 2++ indicated the high quality of the conducted study, and 3 indicated a low level of evidence.

## 3. Results

Based on the PRISMA chart flow, the PubMed, Scopus, and Google Scholar databases yielded 213, 242, and 404 scientific articles, respectively (Figure 1). Subsequently, 21 articles were selected for data extraction and quality assessment based on specific criteria (Table 2). The majority of the analyzed studies used AI to determine the relationship between the mandibular canal and the impacted mandibular third molar (Table 2). The data were extracted from each study according to the author and year, sample number, study design, diagnostic tool, AI application method, comparison to other diagnostic tools, diagnostic imaging technique used for the comparison, and results (Table 3).

Based on the quality assessment using the SIGN method, the average category of the studies was 2+, which indicated that the level of evidence was B, thus implying an adequate methodological quality of the studies (Table 3).

## 4. Discussion

In recent years, several studies have sought to explore the application of AI in diagnosing, predicting, and managing third molar impactions [21,22,41].

The articles selected in this systematic review reveal multifaceted applications of AI in patients with third molar impaction and highlight the most effective and accurate machine learning models, YOLO (versions 3, 5, and 8) and VGGNet (version 16). In a recent study, Lahoud et al. [23] suggested the importance of designing software and programming languages according to the learning and training needs of AI. On the other hand, Ariji et al. [24] indicated that an image editing program is sufficient to differentiate the anatomical structures of the region of origin (third molars) via colored lines.

However, the identification of structures is subject to the observer’s experience and expertise. Hence, the learning of the structures will depend on the knowledge of the human observer. Recent studies have reported the efficacy of semi-supervised learning [25] and deep learning methods [26] in diagnosing impacted third molars using panoramic radiography. Other studies have established clear differences in the accuracy of identifying anatomical structures between those captured on panoramic radiographic images and those captured on CBCT scans [27,28]. CBCT represents a higher cost of treatment and planning and a much higher radiation exposure, which may be unnecessary [42].

### 4.1. Prediction of Third Molar Impaction

Several studies have explored the effectiveness of AI models in predicting third molar impaction [21,41]. These models, often based on machine learning algorithms such as YOLO, RedNet, MobileNet, and CNN, use variables such as age, gender, and measurements from images generated from radiographs or CT scans. Alternatively, few studies have contemplated the demographic characteristics of the sample [29,30]. A retrospective longitudinal study conducted by Chopra et.al [41] on AI-assisted radiographic prediction of lower third molar eruption in two age groups (8–15 with mixed dentition and 16–23 with permanent dentition) concluded that the predictive model for third molar eruption determination was not possible. However, evidence of predicting molar uprighting was strong in the mixed dentition group, and the positive predictive value was 67%. Determining the future horizon of AI in predicting third molar eruption has been deemed difficult [41]. Vranckx et al. [18] investigated the potential eruption of impacted mandibular third molars using a convolutional neural network (CNN) and reported that the AI tool may be used to anticipate the eruption of the third molar quickly and easily during adolescence by predicting the segmentation maps and orientation lines of the molars on panoramic radiographs with accuracy.

The AI-based model has been reported to achieve decent accuracy in helping students and residents [19,21]. However, in cases where the comparison was between an experienced radiologist and a surgeon with AI models, the diagnostic accuracy rates were not that significant [27]. Thus, the accuracy of the results of these models may be overestimated when compared to the diagnostic accuracy of the students and residents [32,42].

### 4.2. Determination of the Position of the Third Molar

The precise location of the third molar is crucial for planning a successful surgical intervention. A recent study by Elborolosy et al. [33] showed that AI can be used as a diagnostic tool to assess the position of the third molar with respect to essential anatomical structures, thus optimizing preoperative strategies and minimizing postoperative complications. Another study that evaluated the diagnostic accuracy of the position of the third molar indicated that the process of recognizing and evaluating the degree of third molar impaction is complex and should not be performed based only on two-dimensional information from panoramic radiographs [43]. Factors such as the density and height of the osseous cortex, the density of the vestibular osseous wall, the apex-coronal and vestibulo-lingual diameter, radicular and coronal morphology, and the Pell–Gregory–Winter classification should be considered to accurately determine the position of the third molar [32]. Some studies have indicated that image segments collected from panoramic radiographs did not consider the dimension of the roots because they generated considerable spatial distortion [42,43]. Only one article considered the Pell–Gregory–Winter classification for the position of the third molar in AI machine learning models [34].

### 4.3. Relationship of the Third Molar with the Inferior Dental Nerve

The proximity of the mandibular third molar to the inferior dental nerve is a critical factor influencing surgical decisions. Some investigators have employed AI to analyze radiographic images and evaluate this relationship [31,35]. AI can aid in accurately identifying this anatomical relationship, facilitating more informed and safer surgical planning [27]. Machine learning models generally allow the detection, classification, and segmentation of the precise inferior dental nerve canal, generating a smooth and accurate clinical workflow [36]. Machine learning models have an image transfer learning technique for recognizing this anatomical structure [27,37]. The recognition of these images in the AI model database will enable greater precision in estimating the positional relationship of the third molar with the dental nerve canal [36]. However, the studies that determined this diagnostic capability did not present quantifiable relationships in length or diameter [22,38]. Alternatively, studies such as those by Vinayahalingam et al. [36] and Gong et al. [44] have emphasized the need to perform segmental imaging of the lower dental nerve canal in a manual and automated manner, using volumetric techniques and 3D reconstructions to obtain a topography of the canal as accurately as possible. AI models can identify dental structures more accurately than other structures, such as nerve canals, root canal anatomy, and soft tissues, possibly due to differences in the hard tissue radiopacity compared to the soft tissue density [36].

### 4.4. Extraction Difficulties

AI has been used to assess the potential difficulties in extracting impacted third molars. Predictive models, such as those described by Kempers et al. [22], have provided an objective assessment to help surgeons better prepare for the varying complexities of extractions. AI models have been used for therapeutic decision-making. One such application is the need to extract impacted molars; although the dentist or another specialist can easily identify and plan the treatment, AI models require prior training coupled with predictive logarithms for estimating the difficulty of tooth extraction [27]. Thanks to semi-automatic image segmentation, AI models can predict complications in impacted third molar extractions. Lee et al. [38] employed AI models using panoramic radiographs to generate learning models that consider the location relationships between the third molar and the lower dental nerve [38]. Limited conjugate databases must be established because the image segments are generated by humans; the AI model should be able to automatically predict the difficulty of extraction and the likelihood of injury to the lower dentition. Thus, AI models will allow dimensional relationships to be established between the third molar and the lower dental nerve. However, the possibility of injury prediction and damage detection may be limited by the amount of data the AI neural network resources can hold.

### 4.5. Strengths and Limitations

This systematic review included three prominent biomedical databases and involved independent reviewers who screened the studies and used the best practices for reporting international guidelines (PRISMA). However, this study has some limitations. Due to the stringent inclusion and exclusion criteria, only a limited number of studies were included. Publications such as reviews, studies investigating other dental aspects, technical reports, and studies in languages other than English and Spanish were excluded; hence, some pertinent literature related to impacted third molars and AI may have been missed. Additional studies comprising more articles are required to validate these findings.

## 5. Conclusions

This review demonstrates the ability of AI neural network models to determine the position, anatomy, and morphology of impacted third molars and predict the eruption of mandibular third molars. Compared to human observers, AI models demonstrate decent performance in determining the morphology, anatomy, and relationship of the impaction with the inferior alveolar nerve canal. However, eruption prediction is still in the developmental stages. Currently, AI research and its applications are in the early stages of development, especially in the field of dentistry. Large data sets are required, and issues such as data privacy need to be handled appropriately. Nonetheless, additional studies estimating the eruption in mixed and permanent dentition are warranted to establish a comprehensive model for identifying, diagnosing, and predicting third molar eruptions and determining the treatment outcomes in the case of impacted teeth. This information will help clinicians make better decisions, resulting in superior treatment outcomes.

## Figures and Tables

**Table 1:** Database search strategy.

Database	Search Strategy
PUBMED	((impacted tooth) OR (mandibular third molar impaction)) AND (artificial intelligence)
SCOPUS	TITLE-ABS-KEY (artificial AND intelligence) AND TITLE-ABS-KEY (impacted AND tooth) OR TITLE-ABS-KEY (mandibular third molar impaction)
Google Scholar	Artificial intelligence AND impacted tooth OR mandibular third molar impaction

**Table 2:** Summary of data extraction from the included studies.

Year and Author	Aims of the Study	Sample	Study Design	Imaging Tool	AI Application Method	Comparison if Applicable	Results
Vranckx et al. (2020) [18]	Prediction of impacted third molar eruption potential	838 patients	Randomized clinical trial	OPG	ResNet-101 learning models	Expert human observer	The accuracy of the angulation measurements of the molars via AI was 79%. The second molar measurements were more accurate than the first molars. Prediction of third molar impaction eruption was reliable.
Orhan et al. (2021) [19]	Impaction location, number, and relation to the inferior alveolar nerve canal	130 molars	Retrospective	CBCT	Deep CNN system	Human observers	A total of 112 impacted teeth, 99 canals, and a relationship between the third molar and maxillary sinus were detected. Accuracy values agreed with those of the human eye.
Celik ME (2022) [21]	Detection of the impaction and proposal of a new method of AI	300 patients	Randomized clinical trial	OPG	Two phases: YOLOV3, Faster RCNN	Human observer	YOLOv3 demonstrated excellent performance in detecting impacted third molars in panoramic images.
Kempers et al. (2023) [22]	Relationship of impaction to the inferior alveolar canal	863 patients	Case–control study	OPG	MobileNet-V2 precision model	Skeletonization algorithm and distance measurement	The MobileNet AI model automatically verified the skeletal, nerve, and dental anatomical relationships in the panoramic radiographs.
Lahoud et al. (2022) [23]	Mandibular canal shape direction and diameter determination	235 patients	Retrospective study	CBCT	Machine learning model of algorithms	Expert observers	The total time for the automated segmentation of the mandibular canal and third molars was 21.2 s, 107 times faster than the manual segmentation performed by the expert.
Ariji et al. (2022) [24]	Relationship of impaction to the inferior alveolar canal and position of impaction	3200 patients	Longitudinal study	OPG	U-Net neural network model	Expert observer; source IA model	The model creation time was 10.5 h. The transfer process was reduced by 90%. The learning models could be replicated and applied to different specimens for the location of impacted third molars.
Kim et al. (2023) [25]	Classification of mandibular third molar	1625 patients	Retrospective	OPG	WideResNet and LaplaceNet model	Expert observer	AI models allowed up to 300 image masks to be collected for diagnosis. Even with fewer image masks, the models had higher accuracy.
Fukuda et al. (2023) [26]	Relationship of impaction to the inferior alveolar canal, and position of impaction	800 patients	Case–control study	CT, CBCT, OPG	Deep learning models	Observer: specialists and residents	The machine learning training process was performed twice, compared with the performances of the observers. The diagnostic accuracy of the residents was 0.85, that of the specialist radiologists was 0.81, and that of the deep learning model was 0.81.
Papasratorn et al. (2023) [27]	Relationship of impaction to the inferior alveolar canal, and position of impaction	1800 patients	Analytical	CBCT	Pre-trained AlexNet, VGG-16, GoogLeNet models	Expert observer	All models recognized the location of the third molar. VGG-16 demonstrated better performance than other trained models.
Lei et al. (2023) [28]	Classification of mandibular third molar	2146 patients	Retrospective	OPG	Model YOLOv5	Expert observer	The introduction of modified AI models increased the accuracy by 3% of the initial model. The amount of time spent on calculations and image processing was significantly reduced.
Joo et al. (2023) [29]	Relationship of impaction to the inferior alveolar canal and position of impaction	5408 patients	Analytical	OPG, CBCT	AI models M3, YOLOv4	Expert opinions	AI models allowed the generation of image masks of the lower dental nerve and third molar. The proposed system verified the classification performance with an improved accuracy of 0.833 using masked images.
Zhu et al. (2021) [30]	Relationship of impaction to the inferior alveolar canal	503 patients	Experimental quantitative	CBCT, OPG	YOLOv4 (MM3-IANnet) deep learning model	CBCT, expert observer	The MM3-INnet demonstrated a higher detection protocol (83%) than the expert dentist (76%) in diagnosing impacted third molars.
Lo Casto et al. (2023) [31]	Diagnostic performance of two conventional neural networks in determining the impaction position	83 patients	Randomized clinical trial	OPG	Two CNNs: ResNet-152 and VGG-19,	Expert (professional) and inexperienced (student) human observer	The student’s diagnostic performance was 62.53%, that of the expert observer was 85.28%, and RestNet achieved 88.86%.
Kwon et al. (2022) [32]	Timing of impaction extraction	724 patients	Cross-sectional study	OPG	CNN + MLP neural network model	Professional experts	A predictive AI model for third molar extraction challenges can be developed with clinical and radiographic data.
Elborolosy et al. (2022) [33]	Difficulty of impacted third molar extraction	2414 patients	Case studies	OPG	VGG-16, MobileNetV2, ResNet50 learning models	Expert observers	The accuracy of the machine learning models was 81% for VGG-16, 79% for MobileNetV2, and 44% for ResNet50. The prediction models and third molar anatomy of VGG1-6 through VGG-19 were comparable to those of the expert.
Takebe et al. (2023) [34]	Relationship of impaction to the inferior alveolar canal and position of impaction	579 patients	Retrospective	OPG	YOLOv3 models	Expert observer	The accuracy rate of the YOLOv3 model was 0.89, while that of the expert professional was 0.6.
Choi et al. (2022) [35]	Relationship of impaction to the inferior alveolar canal	571 patients	Case–control studies	OPG, CBCT	ResNet-50 model of AI	Expert observers	In terms of determining the buccolingual position, expert practitioners demonstrated an accuracy of 69%, and AIN models showed an accuracy of 80%.
Vinayahalingam et al. (2019) [36]	Relationship of impaction to the inferior alveolar canal	81 patients	Randomized clinical trial	OPG	CNN model (M3, IAN)	Panoramic radiography	The CNN segmenters showed defects only in the apical region of the third molar. The training data guide the automated segmentation through AI CNN applications.
Fukuda et al. (2020) [37]	Relationship of impaction to the inferior alveolar canal	600 patients	Case–control study	OPG	Three CNNs	Expert overlooker	The CNN layers determined the storage space and learned parameters. No difference in diagnostic performance was observed between the CNN patches.
Lee et al. (2022) [38]	Prediction of extraction difficulty of the impacted mandibular third molar	5397 patients	Retrospective	OPG	Machine learning model of neural networks	Expert observer	Learning the region of interest (third molar) allowed for the prediction of the difficulty of extraction and the probability of injury to the lower dental nerve in 99% of the cases. An accuracy of 83% was obtained for the prediction of extraction difficulty.
Kwak et al. (2020) [39]	Detection and segmentation of the mandibular canal on CBCT images using various deep learning networks were attempted in this study to investigate the possibility of clinical application	102 patients	Retrospective	OPG	VGGNet, SegNet learning network models	Expert observer	SegNet had an overall accuracy of 0.82, higher than other models without prior tooth segmentation. The learning channels gave higher accuracy in treatment planning.

AI, artificial intelligence; CNN, convolutional neural network; CBCT, cone beam computed tomography; MLP, multilayer perception; OPG, orthopantomogram; M3, lower third molar; IAN, inferior alveolar nerve.

**Table 3:** Summary of the risk of bias assessment.

Selected Article	SIGN Level
Orhan et al. (2021) [19]	2++
Celik (2022) [21]	2+
Lo casto et al. (2023) [31]	2+
Fukuda et al. (2020) [37]	2+
Zhu et al. (2021) [30]	3
Vinayahalingam et al. (2019) [36]	2+
Kwon et al. (2022) [32]	3
Kempers et al. (2023) [22]	2+
Choi et al. (2022) [35]	2+
Elborolosy et al. (2022) [33]	3
Vranckx et al. (2020) [18]	2+
Joo et al. (2023) [29]	2++
Ariji et al. (2022) [24]	2+
Lahoud et al. (2022) [23]	3
Papasratorn et al. (2023) [27]	3
Takebe et al. (2023) [34]	2+
Kim et al. (2023) [25]	2+
Lei et al. (2023) [28]	3
Fukuda et al. (2023) [26]	3
Lee et al. (2022) [38]	3
Kwak et al. (2020) [39]	2+
